# Inflammation mediates the association between furan exposure and the prevalence and mortality of chronic obstructive pulmonary disease: National Health and Nutrition Examination Survey 2013–2018

**DOI:** 10.1186/s12889-024-18442-9

**Published:** 2024-04-15

**Authors:** Di Sun, Yuanying Wang, Jingwei Wang, Nafeisa Dilixiati, Qiao Ye

**Affiliations:** Department of Occupational Medicine and Toxicology, Clinical Center for Interstitial Lung Diseases, Beijing Institute of Respiratory Medicine, Beijing Chao-Yang Hospital, Capital Medical University, 100020 Beijing, China

**Keywords:** Chronic obstructive pulmonary disease, Furan, Inflammation, Prognosis, NHANES

## Abstract

**Background:**

Although extensive research has established associations between chronic obstructive pulmonary disease (COPD) and environmental pollutants, the connection between furan and COPD remains unclear. This study aimed to explore the association between furan and COPD while investigating potential mechanisms.

**Methods:**

The study involved 7,482 adults from the National Health and Nutrition Examination Survey 2013–2018. Exposure to furan was assessed using blood furan levels. Participants were categorized into five groups based on quartiles of log10-transformed blood furan levels. Logistic regression and restricted cubic spline regression models were used to assess the association between furan exposure and COPD risk. Mediating analysis was performed to assess the contribution of inflammation to the effects of furan exposure on COPD prevalence. Cox regression was used to assess the association between furan exposure and the prognosis of COPD.

**Results:**

Participants with COPD exhibited higher blood furan levels compared to those without COPD (*P* < 0.001). Log10-transformed blood furan levels were independently associated with an increased COPD risk after adjusting for all covariates (Q5 vs. Q1: OR = 4.47, 95% CI = 1.58–12.66, *P* = 0.006, *P* for trend = 0.001). Inflammatory cells such as monocytes, neutrophils, and basophils were identified as mediators in the relationship between furan exposure and COPD prevalence, with mediated proportions of 8.73%, 20.90%, and 10.94%, respectively (all *P* < 0.05). Moreover, multivariate Cox regression analysis revealed a positive correlation between log10-transformed blood furan levels and respiratory mortality in COPD patients (HR = 41.00, 95% CI = 3.70–460.00, *P* = 0.003).

**Conclusions:**

Exposure to furan demonstrates a positive correlation with both the prevalence and respiratory mortality of COPD, with inflammation identified as a crucial mediator in this relationship.

## Graphical Abstract

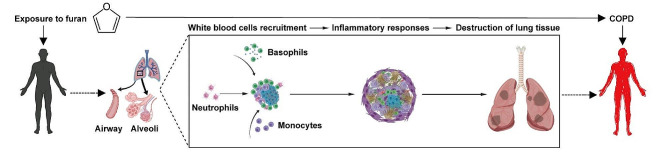

## Introduction

Chronic obstructive pulmonary disease (COPD) is a progressive respiratory disease characterized by persistent airflow limitation and chronic respiratory symptoms [1, 2]. Manifesting as chronic bronchitis and emphysema, COPD often results from prolonged exposure to irritants such as cigarette smoke, air pollution, occupational dust, or chemicals [3, 4]. Globally, COPD poses a significant health burden, emerging as a leading cause of morbidity and mortality [5]. A crucial aspect in addressing COPD involves identifying risk factors and proactively implementing interventions to mitigate the prevalence and mortality of the disease [2].

Furan, with the chemical formula C_4_H_4_O, is a heterocyclic compound featuring a five-membered ring composed of four carbon atoms and one oxygen atom [6]. Its widespread application extends to lacquer formation, resin solvents, and contributions to agricultural chemicals, stabilizers, and pharmaceuticals [7]. Furthermore, furan serves as a significant heat-induced dietary contaminant, being detected in a diverse array of heat-processed foods [8]. While some studies have suggested that exposure to furan may cause alterations in lung pathology similar to COPD, characterized by inflammatory cells infiltration, alveolar structure destruction, and the development of emphysema [9, 10], comprehensive research is lacking to clarify the association between furan and COPD, highlighting the necessity for further investigation in this area.

Inflammatory cells, notably white blood cells (WBCs), are major pro-inflammatory contributors, pivotal in initiating, regulating, and resolving inflammatory responses [11]. WBCs serve as reliable indicators of systemic inflammation [12, 13], routinely evaluated through well-standardized automated methods, which are both cost-effective and highly accurate.

The objective of this study is to elucidate the relationship between furan exposure and the prevalence and prognosis of COPD, utilizing data from the National Health and Nutrition Examination Survey (NHANES). Additionally, the study aims to explore potential inflammatory mechanisms underlying this association by examining WBCs.

## Materials and methods

### Study design and participants

NHANES, a cross-sectional survey of national representation conducted by the National Center for Health Statistics (NCHS), has been conducted annually since 1999. This comprehensive survey involves approximately 5,000 individuals who undergo interviews, health examinations, and laboratory tests. All participants provided written informed consent, and the survey protocol received approval from the NCHS Research Ethics Review Board [14]. The dataset, publicly accessible on the NHANES website (http://www.cdc.gov/nchs/nhanes/index.htm), formed the basis of our study, encompassing 29,400 participants across three cycles (2013–2014, 2015–2016, and 2017–2018) [15, 16].

**Fig. 1:**
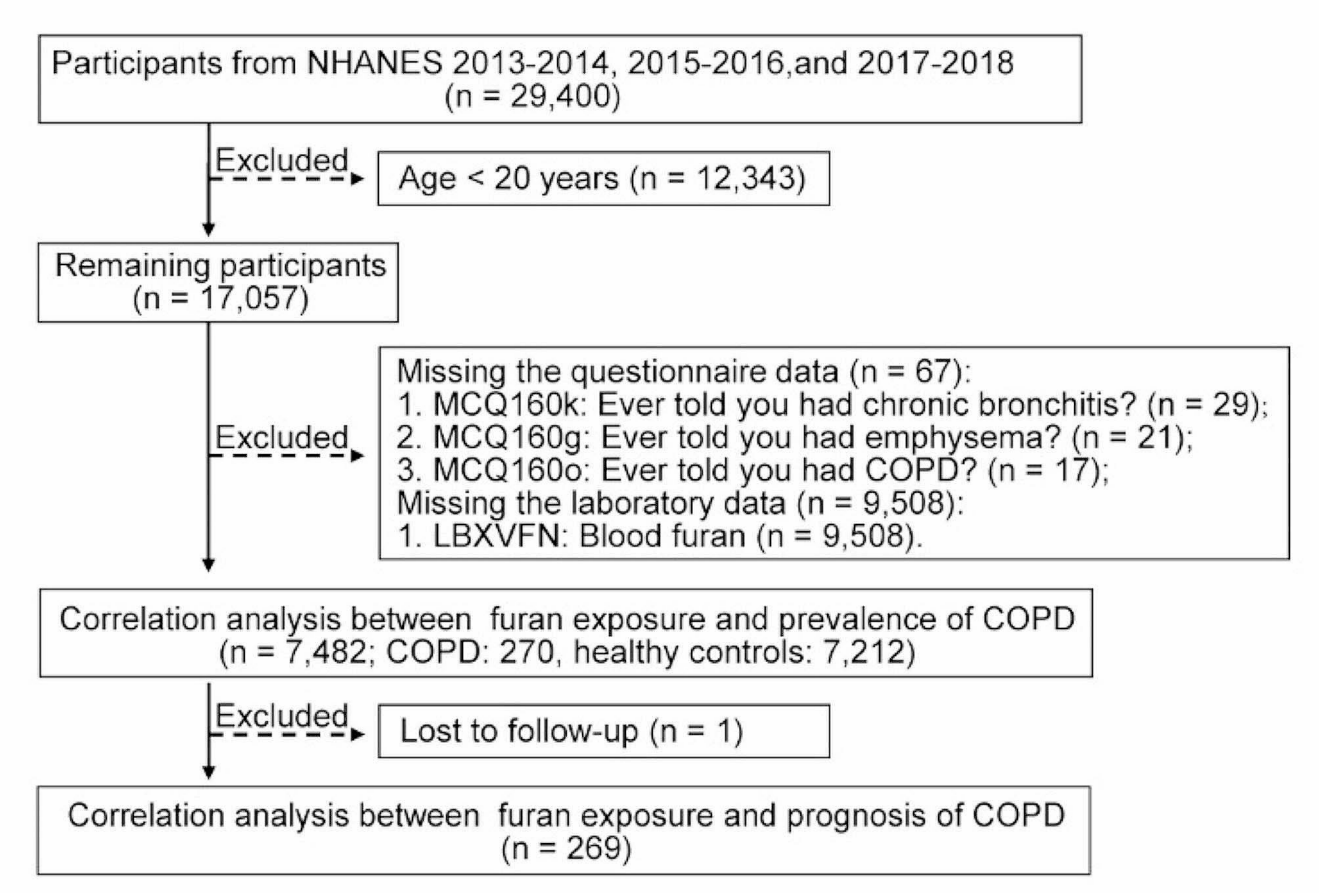
Flowchart of patient screening and selection for this study. Abbreviations: NHANES, National Health and Nutrition Examination Survey; COPD, chronic obstructive pulmonary disease

After merging databases, individuals under the age of 20 (*n* = 12,343), those lacking questionnaire data (*n* = 67), and individuals with missing laboratory data (*n* = 9,508) were excluded. Consequently, 7,482 participants met the criteria for further analysis, with no duplicated cases identified (Fig. 1).

### COPD

COPD was defined based on a self-reported physician diagnosis. Participants were identified as having COPD if they confirmed a positive response to the specific query: “Ever told you had COPD?”. Additionally, non-emphysematous COPD was defined for COPD patients who responded “No” to the question “Ever told you had emphysema?“. These inquiries were part of a standardized medical condition questionnaire administered during individualized interviews.

### Furan measurement

Monitoring blood furan levels emerges as a valuable method for extracting insights into exposure levels and internal dosage [7]. To achieve this objective, a systematic collection of whole blood samples was conducted, followed by the development of an automated analytical method using capillary gas chromatography (GC) and mass spectrometry (MS) with selected-ion monitoring (SIM) detection and isotope-dilution. The method demonstrated efficacy in detecting furan within modest blood samples and exhibited a notably low detection threshold, making it well-suited for application in the general population. Rigorous measures were implemented to minimize contamination sources, uphold laboratory efficiency, and ensure result quality, including the periodic reexamination of 2% of all specimens.

### Data collection

Drawing from previous research [17], we incorporated various covariates into our analysis to explore potential factors influencing COPD. The gathered covariate data encompassed: (1) demographics data: age, gender, marital status, ethnicity, educational attainment, and past-year alcohol drinking; (2) examination data: body mass index (BMI); and (3) laboratory data: serum cotinine. Serum cotinine, a primary metabolite in nicotine biotransformation, served as a biomarker for smoking status [18].

In addition, supplementary data were collected, including: (1) clinical manifestations: chronic bronchitis and emphysema; (2) comorbidities: hypertension, diabetes mellitus (DM), coronary heart disease (CHD), and cancer/malignancy; and (3) the actual count of WBCs (1,000 cells/uL): lymphocytes, monocytes, neutrophils, eosinophils, and basophils.

### Outcome

The primary outcome of our study focused on respiratory mortality, encompassing chronic lower respiratory diseases (J40 - J47), influenza, and pneumonia (J10 - J18). We determined mortality status and cause of death using NHANES-linked National Death Index public access files through December 31, 2019.

### Statistical analysis

The statistical analyses adhered to CDC analytical reporting guidelines, specifically designed for the nuanced analysis of NHANES data [15, 16]. We considered concealed variance and implemented the recommended weighting scheme, utilizing individual sample weights based on six years of VOC subsample weight (WTSVOC2Y), as per NHANES recommendations.

Variables with missing data below 25%, such as marital status, educational attainment, cotinine, and BMI, were treated as dummy variables to mitigate the reduction in sample size. Continuous variables with non-normal distributions were presented as the median (25th, 75th) and assessed using the Wilcoxon rank-sum test for complex survey samples. Categorical variables were expressed as absolute values (n) and percentages (%) and analyzed through the chi-square test (Rao & Scott’s second-order correction).

To address the skewed distribution of furan levels, a log10 transformation was applied to normalize the distributions. Moreover, participants were categorized into five groups based on the quartiles of log10-transformed blood furan levels: Q1 group (below the detectable threshold [log10-transformed blood furan levels < -1.61 ng/mL], *n* = 6,219), Q2 group (-1.61 ng/mL ≤ log10-transformed blood furan levels < -1.39 ng/mL, *n* = 303), Q3 group (-1.39 ng/mL ≤ log10-transformed blood furan levels < -1.19 ng/mL, *n* = 322), Q4 group (-1.19 ng/mL ≤ log10-transformed blood furan levels < -0.99 ng/mL, *n* = 317), and Q5 group (log10-transformed blood furan levels ≥ -0.99 ng/mL, *n* = 321).

To explore the dose-response association between log10-transformed blood furan levels and COPD risk, we employed a restricted cubic spline (RCS) analysis. Univariate and multivariate logistic regression analyses were utilized to investigate the association between furan levels and COPD prevalence, considering covariates. Initially, the crude model, without any covariate adjustments, was employed. Subsequently, Model I incorporated adjustments for cotinine, while Model II included adjustments for cotinine, age, gender, marital status, ethnicity, educational attainment, past-year alcohol drinking, and BMI. For multivariate survival analysis, a Cox regression model was used.

Additionally, multivariable linear regression models were used to examine the correlation between log10-transformed blood furan levels, COPD prevalence, and inflammatory cells. Furthermore, causal mediation analysis was performed using the R package “mediation” (version 4.5.0) with 1,000 simulations [19]. A model-based inference approach estimated the average causal mediation effect (ACME), average direct effect (ADE), and average total effect [19, 20]. The proportion of the effect of the exposure on the outcome passing through the mediator was evaluated by dividing the ACME by the total effect (ACME + ADE).

All statistical analyses were executed using R (version 4.2.2) with RStudio (version 2022.07.2 Build 576). The predetermined threshold for statistical significance was established at *P*-values below 0.05.

## Results

### Baseline characteristics of participants

As illustrated in Fig. 1, a total of 7,482 participants were enrolled in the present study, sourced from the NHANES database covering the years 2013 to 2018. The weighted prevalence of COPD was determined to be 3.35%, comprising 270 participants diagnosed with COPD and 7,212 participants without COPD. Notably, 44.5% of COPD patients clinically presented with chronic bronchitis, while 37.1% manifested emphysema. As shown in Table 1, statistically significant differences were observed in age, marital status, ethnicity, educational attainment, serum cotinine, clinical manifestations, and comorbidities (all *P* < 0.05). However, no significant differences were noted in gender, past-year alcohol drinking, and BMI (all *P* > 0.05). In comparison to the healthy controls, there is a significant increase in the proportion of COPD patients in Q2 group, Q3 group, Q4 group, and Q5 group (*P* < 0.001).

**Table 1:** Demographics and clinical characteristics of participants

Characteristics	All (*n* = 7,482)	Healthy controls (*n* = 7,212)	COPD (*n* = 270)	P-value
Age (years)	47 (33, 61)	47 (33, 60)	63 (57, 72)	< 0.001
Gender, n (%)				
Female	3,849 (51.5%)	3,724 (51.5%)	125 (51.6%)	
Male	3,633 (48.5%)	3,488 (48.5%)	145 (48.4%)	0.984
Marital status, n (%)				
Married	3,826 (53.9%)	3,703 (53.9%)	123 (55.0%)	
Never	1,373 (18.5%)	1,355 (19.0%)	18 (5.0%)	
Other	2,279 (27.6%)	2,151 (27.1%)	128 (40.0%)	< 0.001
Ethnicity, n (%)				
Mexican American	1,116 (8.9%)	1,102 (9.2%)	14 (2.1%)	
Non-Hispanic White	2,800 (63.9%)	2,617 (63.3%)	183 (80.6%)	
Non-Hispanic Black	1,534 (11.1%)	1,499 (11.3%)	35 (5.7%)	
Other Hispanic	800 (6.4%)	790 (6.5%)	10 (1.8%)	
Other Race	1,232 (9.7%)	1,204 (9.7%)	28 (9.8%)	< 0.001
Educational attainment, n (%)				
Middle school or lower	1,606 (13.6%)	1,527 (13.4%)	79 (20.9%)	
High school	1,717 (23.6%)	1,635 (23.3%)	82 (32.1%)	
College or more	4,153 (62.7%)	4,045 (63.3%)	108 (47.0%)	0.004
Serum cotinine (ng/mL)	0.03 (0.01, 3.57)	0.03 (0.01, 2.07)	3.96 (0.02, 258.20)	< 0.001
Past-year alcohol drinking, n (%)				
Never	1,579 (19.8%)	1,550 (20.0%)	29 (12.2%)	
1–3 drinks/day	3,408 (63.6%)	3,301 (63.4%)	107 (69.4%)	
≥ 4 drinks/day	956 (16.7%)	925 (16.6%)	31 (18.4%)	0.171
BMI (kg/m^2^)	28.5 (24.7, 33.1)	28.4 (24.7, 33.1)	29.1 (25.5, 33.8)	0.223
Clinical manifestations, n (%)				
Chronic bronchitis	425 (5.8%)	314 (4.5%)	111 (44.5%)	< 0.001
Emphysema	140 (1.6%)	44 (0.4%)	96 (37.1%)	< 0.001
Comorbidities, n (%)				
Hypertension	721 (10.7%)	658 (10.2%)	63 (26.7%)	< 0.001
Diabetes mellitus	1,040 (10.4%)	969 (9.9%)	71 (24.9%)	< 0.001
Coronary heart disease	296 (3.6%)	235 (2.9%)	61 (23.8%)	< 0.001
Cancer or malignancy	721 (10.7%)	658 (10.2%)	63 (26.7%)	< 0.001
Blood furan, n (%)				
Q1 group	6,219 (84.0%)	6,061 (84.7%)	158 (63.7%)	
Q2 group	303 (4.0%)	287 (3.9%)	16 (5.2%)	
Q3 group	322 (3.8%)	296 (3.7%)	26 (6.4%)	
Q4 group	317 (3.9%)	289 (3.7%)	28 (7.0%)	
Q5 group	321 (4.3%)	279 (3.8%)	42 (17.6%)	< 0.001

Continuous variables are presented as median (25th, 75th) for continuous variables. Categorical variables are expressed as unweighted frequency counts and weighted percentages. Abbreviations: COPD, chronic obstructive pulmonary disease; BMI, body mass index

### Furan exposure and COPD prevalence

**Fig. 2:**
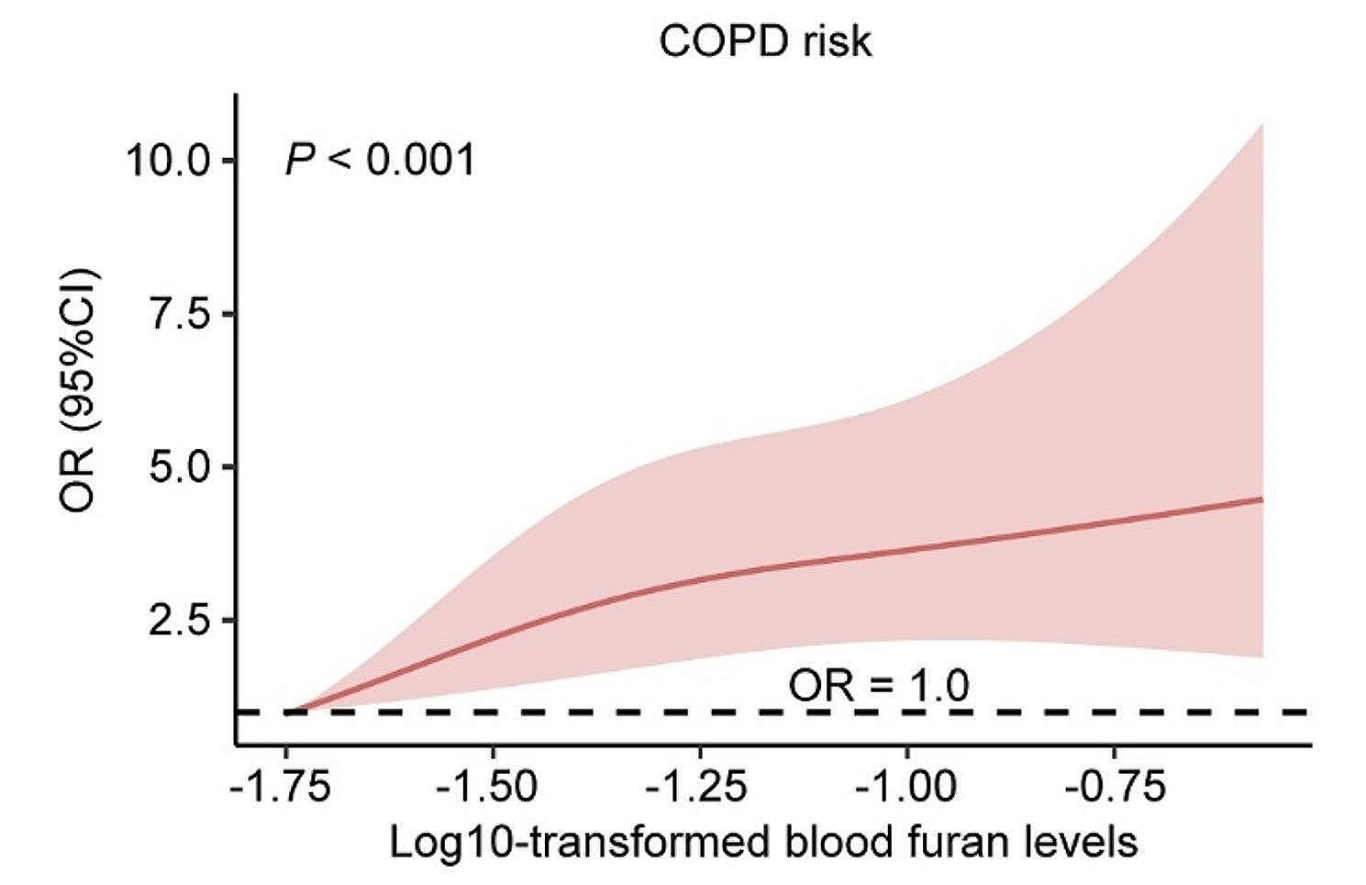
Restricted cubic spline plot. Abbreviations: COPD, chronic obstructive pulmonary disease; OR, odd ratio; CI, confidence interval

The RCS model exhibited a positive association between the log10-transformed blood furan levels and the risk of COPD, even after adjusting for all covariates (*P* < 0.001). With an increase in log10-transformed blood furan levels, there is a concurrent rise in the risk of COPD (Fig. 2).

As presented in Table 2, logistic regression analysis demonstrated a correlation between continuous log10-transformed blood furan levels and an increased risk of COPD (all *P* < 0.05). In comparison to the Q1 group, participants in the Q5 group exhibited a higher risk of COPD in all three models (crude model: OR = 6.11, 95% CI = 3.71–10.05, *P* < 0.001, *P* for trend < 0.001; model I: OR = 3.35, 95% CI = 1.85–6.07, *P* < 0.001, *P* for trend < 0.001; model II: OR = 4.47, 95% CI = 1.58–12.66, *P* = 0.006, *P* for trend = 0.001). Additionally, furan exposure also demonstrated a positive correlation with the prevalence of non-emphysematous COPD (all *P* < 0.05; Table 3).

**Table 2:** Logistic regression analysis of log10-transformed blood furan levels for the risk of COPD

Blood furan	Crude Model		Model I		Model II	
	OR (95% CI)	P-value	OR (95% CI)	P-value	OR (95% CI)	P-value
Log10-transformed blood furan	6.00 (3.58–10.05)	< 0.001	3.14 (1.64–5.99)	< 0.001	4.70 (1.58–13.94)	0.007
Q1 group	Reference		Reference		Reference	
Q2 group	1.75 (0.78–3.89)	0.168	1.40 (0.61–3.22)	0.417	2.09 (0.70–6.28)	0.181
Q3 group	2.29 (1.32–3.99)	0.004	1.54 (0.85–2.80)	0.147	3.10 (1.29–7.41)	0.013
Q4 group	2.50 (1.32–4.74)	0.006	1.54 (0.75–3.14)	0.230	3.03 (1.13–8.08)	0.028
Q5 group	6.11 (3.71–10.05)	< 0.001	3.35 (1.85–6.07)	< 0.001	4.47 (1.58–12.66)	0.006
*P* for trend	< 0.001		< 0.001		0.001	

The crude model lacked adjustments for covariates. Model I incorporated cotinine adjustments. Model II included adjustments for cotinine, age, gender, marital status, ethnicity, educational attainment, past-year alcohol drinking, and BMI. Abbreviations: COPD, chronic obstructive pulmonary disease; OR, odd ratio; CI, confidence interval

**Table 3:** Logistic regression analysis of log10-transformed blood furan levels for the risk of non-emphysematous COPD

Blood furan	Crude Model		Model I		Model II	
	OR (95% CI)	P-value	OR (95% CI)	P-value	OR (95% CI)	P-value
Log10-transformed blood furan	5.74 (3.30–9.98)	< 0.001	4.20 (2.46–7.18)	< 0.001	6.93 (2.75–17.45)	< 0.001
Q1 group	Reference		Reference		Reference	
Q2 group	1.18 (0.50–2.76)	0.697	1.05 (0.45–2.43)	0.916	1.54 (0.48–4.97)	0.455
Q3 group	2.29 (1.11–4.75)	0.027	1.84 (0.87–3.90)	0.106	3.69 (1.21–1.12)	0.023
Q4 group	1.73 (0.74–4.05)	0.201	1.32 (0.56–3.13)	0.512	3.08 (1.20–7.90)	0.021
Q5 group	6.14 (3.57–10.56)	< 0.001	4.41 (2.65–7.35)	< 0.001	7.21 (2.92–17.82)	< 0.001
*P* for trend	< 0.001		< 0.001		< 0.001	

The crude model lacked adjustments for covariates. Model I incorporated cotinine adjustments. Model II included adjustments for cotinine, age, gender, marital status, ethnicity, educational attainment, past-year alcohol drinking, and BMI. Abbreviations: COPD, chronic obstructive pulmonary disease; OR, odd ratio; CI, confidence interval

### Furan exposure, COPD prevalence, and inflammation

The association between WBCs and inflammation is well-established, encompassing neutrophils, lymphocytes, monocytes, eosinophils, and basophils [12, 21]. A significantly positive association between log10-transformed blood furan levels and lymphocyte, monocytes, neutrophils, and basophils (all *P* < 0.05; Table 4). However, no correlation was observed with eosinophils (*β* = 1.29 × 10^− 2^, 95% CI = -2.88 × 10^− 2^– 5.46 × 10^− 2^, *P* = 0.533).

**Table 4:** Linear regression of log10-transformed blood furan levels and COPD risk with WBCs

WBCs (1000 cells/uL)	Furan			COPD	
	β (95%CI)	P-value		β (95%CI)	P-value
Lymphocyte	3.80 × 10^− 1^ (2.00 × 10^− 1^– 5.61 × 10^− 1^)	< 0.001		-3.82 × 10^− 4^ (-1.06 × 10^− 3^– 3.01 × 10^− 4^)	0.264
Monocytes	7.19 × 10^− 2^ (3.84 × 10^− 2^– 1.05 × 10^− 1^)	< 0.001		4.93 × 10^− 2^ (1.82 × 10^− 2^– 8.04 × 10^− 2^)	0.003
Neutrophils	8.78 × 10^− 1^ (5.69 × 10^− 1^– 1.19 × 10^0^)	< 0.001		5.81 × 10^− 3^ (2.37 × 10^− 3^– 9.26 × 10^− 3^)	0.002
Eosinophils	1.29 × 10^− 2^ (-2.88 × 10^− 2^– 5.46 × 10^− 2^)	0.533		4.11 × 10^− 2^ (-1.79 × 10^− 2^– 1.00 × 10^− 1^)	0.166
Basophils	1.81 × 10^− 2^ (9.86 × 10^− 3^– 2.64 × 10^− 2^)	< 0.001		1.95 × 10^− 1^ (9.84 × 10^− 2^– 2.91 × 10^− 1^)	< 0.001

Adjusted for cotinine, age, gender, marital status, ethnicity, educational attainment, past-year alcohol drinking, and BMI. Abbreviations: WBCs, white blood cells; COPD, chronic obstructive pulmonary disease; BMI, body mass index; CI, confidence interval

Additionally, COPD exhibited a significantly positive association with monocytes, neutrophils, and basophils (all *P* < 0.05; Table 4). However, no correlation was observed with lymphocytes and eosinophils (all *P* > 0.05; Table 4). These results underscore the association between furan or COPD and inflammation.

### Inflammation involved in the effects of furan on COPD

Mediation analysis was employed to explore the mediating influence of inflammation on the relationship between furan exposure and COPD prevalence. The findings presented in Table 5 indicate that monocytes, neutrophils, and basophils emerged as significant mediators in the associations between furan exposure and COPD prevalence. The mediated proportions for monocytes, neutrophils, and basophils were 8.73% (*P* = 0.026), 20.90% (*P* = 0.020), and 10.94% (*P* = 0.024), respectively. These results contribute to a more comprehensive understanding of the interplay between furan exposure, inflammation, and COPD prevalence.

**Table 5:** Mediating role of WBCs in the association between furan exposure and COPD risk

WBCs (1000 cells/uL)	ACME	ADE	Average total effect	Mediated proportion (%)	P-value
	β (95%CI)	β (95%CI)	β (95%CI)		
Monocytes	2.93 × 10^− 3^ (1.22 × 10^− 3^, 0.01)^***^	3.07 × 10^− 2^ (6.66 × 10^− 3^, 0.09)^*^	3.36 × 10^− 2^ (1.08 × 10^− 2^, 0.10)^*^	8.73	0.026
Neutrophils	7.02 × 10^− 3^ (1.86 × 10^− 3^, 0.01)^***^	2.66 × 10^− 2^ (5.01 × 10^− 3^, 0.09)^*^	3.36 × 10^− 2^ (1.11 × 10^− 2^, 0.10)^*^	20.90	0.020
Basophils	3.68 × 10^− 3^ (1.03 × 10^− 3^, 0.01)^***^	2.99 × 10^− 2^ (3.93 × 10^− 3^, 0.09)^*^	3.36 × 10^− 2^ (6.67 × 10^− 3^, 0.10)^*^	10.94	0.024

Adjusted for cotinine, age, gender, marital status, ethnicity, educational attainment, past-year alcohol drinking, and BMI. Abbreviations: WBCs, white blood cells; COPD, chronic obstructive pulmonary disease; ACME, average causal mediation effect; ADE, average direct effect; BMI, body mass index; CI, confidence interval. ^*^ 0.01 ≤ *P* < 0.05, ^***^P < 0.001

### Furan exposure and respiratory mortality of COPD

To further explore the association between furan exposure and the respiratory mortality of COPD, Cox regression analysis was conducted. With one patient lost to follow-up and excluded (Fig. 1), among 269 COPD patients, 10 succumbed to respiratory diseases, leading to a weighted respiratory mortality of 3.91%. After adjusting for cotinine, age, BMI, and comorbidities, the Cox regression analysis uncovered a positive correlation between log10-transformed blood furan levels and respiratory mortality in COPD patients (hazard ratio [HR] = 41.00, 95% CI = 3.70–460.00, *P* = 0.003).

## Discussion

Our study, using NHANES data from 2013 to 2018, was the first to explore the complex connections between furan exposure and COPD prevalence and prognosis in adults. A positive link between log10-transformed blood furan levels and the prevalence and prognosis of COPD were found. Moreover, inflammatory cells like monocytes, neutrophils, and basophils might play a role in mediating this association. This finding contributes new insights into environmental risk factors for COPD, informing public health strategies and deepening our understanding of the intricate interplay between environmental exposures and respiratory health.

COPD, characterized as a persistent respiratory inflammation, is an irreversible and preventable disease [22]. Despite a decrease in the COPD death rate from 1990 to 2015 [23, 24], it is still projected to become the third leading cause of global mortality by 2030 [25]. Furan, often underestimated as a chemical environmental risk factor for COPD, is found in chemical fumes and heat-processed foods [4, 6, 26–29]. In this study, we discovered a positive correlation between the log10-transformed blood furan levels and the risk of COPD. Non-emphysematous COPD, acknowledged as a significant subtype of COPD with distinct rates of disease progression and mortality [30], is also linked to log10-transformed blood furan levels. Crucially, these results were obtained while adjusting for covariates. This indirectly suggests that furan, as a common environmental pollutant, may serve as one of the risk factors for causing COPD.

While inhalation serves as the main pathway for human exposure to furan [31], previous studies on furan toxicity have mainly concentrated on its adverse effects on the liver, with minimal consideration given to its impact on the respiratory system [32–34]. Previous studies have associated inhaling furan with damage to the respiratory system, characterized by the recruitment of inflammatory cells and the destruction of alveolar walls [9, 10, 35–40]. Furthermore, club cell degeneration is observed, marked by cell swelling and cytoplasmic vacuolation, along with necrosis, leading to widespread cell sloughing and multifocal bronchiolar denudation, resulting in occlusion in the lumen of the lower respiratory tract [10, 41]. Bas et al.‘s study suggested that consuming furan through food could also result in alveolar wall rupture, the accumulation of inflammatory cells, and lung tissue changes resembling emphysema [9]. These studies collectively demonstrate that furan, whether ingested orally or inhaled, can lead to lung damage, causing pathological manifestations similar to COPD [42]. However, existing studies have primarily concentrated on short-term exposure to relatively high doses of furan. Further research, particularly investigating the implications of long-term exposure to lower doses, is crucial to establishing a comprehensive understanding of the relationship between furan and COPD.

In this study, we identified inflammatory cells like monocytes, neutrophils, and basophils as crucial mediators in elucidating the connections between furan exposure and the prevalence of COPD. Prior studies suggest that most COPD patients exhibit elevated levels of neutrophils and macrophages in sputum, indicating increased secretion of chemotactic mediators for neutrophils and monocytes in the lungs [43, 44]. Exposure to furan is associated with the recruitment of neutrophils in lung tissue, causing damage to alveoli and airway obstruction, potentially contributing to the development of COPD [9, 10, 41]. Lung macrophages, partly derived from blood monocytes, play a crucial role as immune effector cells in responding to inhaled chemicals, contributing to both innate and adaptive immune responses [45]. Macrophages possess a wide array of functional properties, including phagocytosis, material processing, and signaling mediator production, significantly impacting the occurrence and progression of COPD [4, 45]. Furthermore, some studies have confirmed that chronic airway inflammation in COPD is associated with basophils in both central airways (bronchi) and distal lung compartments [44]. In recent experimental models, basophils have been identified as having a role in emphysema development through IL-4-mediated generation of MMP-12-producing macrophages [46]. Hence, it is reasonable to speculate that inflammation may represent a potential mechanism underlying furan-related COPD.

This study further discerned that exposure to furan elevates the risk of respiratory mortality in patients with COPD. Consistent with earlier investigations, such exposure has been linked to pulmonary injury, including the potential development of respiratory failure, ultimately leading to mortality [9, 10, 37, 41]. The cumulative findings from these studies underscore the imperative for patients with COPD to refrain from furan exposure, thereby augmenting life expectancy.

Some limitations persist in the present study. Firstly, it employs a cross-sectional design, impeding the establishment of causal relationships when exploring the connection between furan exposure and the prevalence of COPD. Secondly, the diagnosis of COPD relies on self-reporting rather than more definitive methods such as pulmonary function testing, which could introduce recall bias. Thirdly, furan absorption can occur through both digestive and respiratory pathways, yet this study fails to specify the precise conditions of exposure. Fourthly, despite childhood respiratory infections being a recognized risk factor for COPD, the lack of relevant data in NHANES prevented this study from adjusting this variable as a covariate. Finally, the absence of multi-pollutant adjustment and exposure misclassification analysis in this study raises the possibility of distorting the association between exposure and documented outcomes.

## Conclusions

In summary, our study suggests a positive association between furan exposure and the prevalence and respiratory mortality of COPD. Monocytes, neutrophils, and basophils have been identified as significant mediators, emphasizing the role of inflammation in this connection. The implication is that reducing exposure to furan in the environment could potentially lower the incidence of COPD and improve the prognosis for COPD patients. However, further large-scale prospective cohort studies are warranted to confirm these findings.

## Data Availability

The datasets used for these analyses are publicly available (https:// www.cdc.gov/nchs/nhanes/index.htm). The code will be provided as required.

## Abbreviations

COPD: Chronic obstructive pulmonary disease
NHANES: National Health and Nutrition Examination Survey
DM: Diabetes mellitus
CHD: Coronary heart disease
WBCs: White blood cells
RCS: Restricted cubic spline
OR: Odds ratio
CI: Confidence interval
HR: Hazard ratio
